# Autoantibodies Targeting the Hypothalamic-Pituitary-Ovarian Axis in Polycystic Ovary Syndrome: Emerging Key Players in Pathogenesis?

**DOI:** 10.3390/ijms26094121

**Published:** 2025-04-26

**Authors:** Nicole Akpang, Jakub Kwiatkowski, Lucja Zaborowska, Artur Ludwin

**Affiliations:** 1 1st Department of Obstetrics and Gynecology, Medical University of Warsaw, 02-015 Warsaw, Poland; 2 Doctoral School of Medical and Health Sciences, Jagiellonian University Collegium Medicum, 31-530 Cracow, Poland

**Keywords:** polycystic ovary syndrome, PCOS, antibodies, autoantibodies, autoimmunity, pathogenesis, hypothalamic-pituitary-ovarian axis

## Abstract

Polycystic ovary syndrome (PCOS) is a common female endocrinopathy associated with reproductive and metabolic abnormalities. PCOS is characterized by complex pathogenesis and pathophysiology. Its multifactorial etiology and heterogeneous presentation make effective treatment difficult. Endocrine abnormalities in PCOS create a vicious cycle of overriding dysfunction involving the hypothalamic-pituitary-ovarian (HPO) axis. Most research has primarily focused on identifying genetic, epigenetic, or immunological factors underlying PCOS. In recent years, new reports have emerged on the possible involvement of antibodies directed against HPO axis components in the development of PCOS. Some of these have been shown to be able to interfere with hormone receptors or receptor binding by targeting the key domains for their function. However, the evidence is heterogeneous and challenging to interpret, given the overall predisposition to high levels of various autoantibodies found in women with PCOS. This review focuses on autoantibodies affecting the HPO axis in PCOS and their potential role in the pathogenesis of PCOS. The authors discuss PCOS as a potential antibody-mediated autoimmune disease in light of recent reports on its possible pathogenesis.

## 1. Introduction

Polycystic ovary syndrome (PCOS) is a common gynecological and endocrinological disorder affecting women of reproductive age, characterized by polycystic ovarian morphology, hyperandrogenism, irregular menstrual cycles, and anovulation [1]. It is frequently accompanied by obesity, insulin resistance, and an increased risk of cardiovascular and autoimmune diseases [2,3]. According to the widely used Rotterdam criteria, PCOS is defined by the presence of two of the following three criteria: oligo-anovulation, hyperandrogenism, and polycystic ovaries [4]. Additionally, serum anti-Müllerian hormone (AMH) can be used for defining polycystic ovarian morphology (PCOM) in adults, especially if transvaginal ultrasound is not feasible [5]. This condition poses both diagnostic and therapeutic challenges. In order to diagnose PCOS, it is essential to exclude other potential causes of the observed clinical features, including thyroid dysfunction, hyperprolactinemia, non-classical congenital adrenal hyperplasia (NCAH), androgen-secreting tumor, Cushing’s syndrome, and acromegaly [6]. Not only is it a diagnosis of exclusion, but also, its exact pathogenesis remains unclear [7]. There is no specific drug for the treatment of PCOS. Current dietary and lifestyle recommendations include a 5% weight reduction, a balanced diet providing 40% of energy from carbohydrates, 30% from fats, and 30% from proteins, and a regular exercise regimen [8,9]. Pharmacological approaches typically involve combined oral contraceptives, progestins, antiandrogenic drugs, metformin, and ovulation induction, typically attained with clomiphene citrate [9]. However, available treatment methods are used off-label and do not address the underlying cause of the disorder, highlighting the urgent need for novel pharmacological solutions specifically designed for the PCOS population [10]. To achieve this, we must first gain a deeper understanding of the molecular mechanisms governing this condition and driving its heterogeneity. It is well established that various factors may contribute to the development of PCOS. These include epigenetic mechanisms, exposure to endocrine-disrupting chemicals, diet, oxidative stress, inflammation, or obesity [9]. What do these factors trigger, and are we able to stop this process? An increased frequency of pulsatile gonadotropin-releasing hormone (GnRH) secretion seems to be one of the most important mechanisms that disrupt the hypothalamic–pituitary–ovarian (HPO) axis in patients with PCOS [11]. As a consequence, researchers have observed an elevated ratio of luteinizing hormone (LH) to follicle-stimulating hormone (FSH), low progesterone levels, and relative hyperestrogenism [12]. In recent years, there has been growing evidence suggesting the presence of autoantibodies against components of the HPO axis and their potential role in the pathogenesis of PCOS [13,14].

## 2. PCOS Pathogenesis and Pathophysiology

### 2.1. Factors Contributing to PCOS Etiology

**Figure 1:**
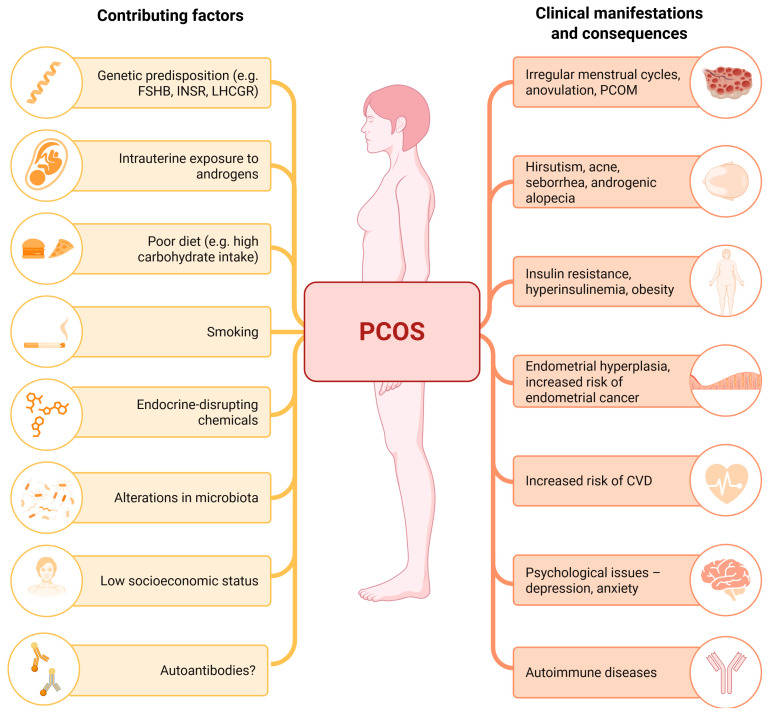
Polycystic ovary syndrome (PCOS) is a complex endocrine disorder with multifactorial causes, which include a combination of genetic, hormonal, metabolic, and environmental factors. The interplay of these causes leads to the diverse clinical consequences and symptoms observed in individuals with PCOS. CVD—cardiovascular disease; FSHB—beta subunit of FSH; INSR—insulin receptor; LHCGR—luteinizing hormone/choriogonadotropin receptor; PCOM—polycystic ovarian morphology. Created with Biorender.com.

The precise etiology underlying the development of PCOS is not fully understood and requires further investigation. The challenges associated with PCOS pathogenesis are further intensified by the substantial heterogeneity of the condition, both in terms of disease features and its multifactorial origins. Numerous diverse causes may contribute to hormonal imbalances that drive the development of PCOS, including (i) genetic predisposition, (ii) disturbances during intrauterine development, or (iii) environmental exposures occurring later in life [15] (Figure 1).

#### 2.1.1. Genetic Factors

PCOS is highly heritable as evidenced by the familial clustering of cases. A Dutch study reported that the occurrence rate of PCOS among monozygotic twin sisters was nearly twice as high as that among dizygotic twins (0.71 vs. 0.38) [16]. Genome-wide association studies (GWASs) identified approximately 20 common susceptibility variants for PCOS in Chinese and European cohorts [17]. Among the candidate genes are receptors for luteinizing hormone/choriogonadotropin (LHCGR), FSH (FSHR), insulin (INSR), and beta subunit of FSH (FSHB) as well as loci associated with the development of type 2 diabetes (THADA, HMGA2) and type 1 diabetes (RAB5B, SUOX, ERBB3), also involved in ovarian follicle development, androgen biosynthesis, and metabolic regulation [12]. Metabolic factors, including body mass index (BMI) and insulin resistance, have been shown to exhibit high heritability among sisters of women with PCOS. Moreover, individual hormonal components of PCOS (testosterone, dehydroepiandrosterone sulfate, sex hormone-binding globulin) are also highly heritable.

#### 2.1.2. Intrauterine and Perinatal Factors

There is compelling evidence that the intrauterine environment may influence PCOS development. In a study on the heritability of fasting dysglycemia in women with PCOS, it was found that the maternal impact was significantly higher than the paternal impact [18], suggesting a potential involvement of intrauterine programming mechanisms and epigenetics. It has been shown that fetal exposure to elevated androgen levels increases the risk of developing PCOS through epigenetic programming [19]. A correlation between the overexposure of the fetus to androgens and the future development of a PCOS-like phenotype was shown in animal studies [20,21]. Additionally, pregnant women with PCOS exhibit elevated levels of circulating androgens and AMH [22,23]. It remains uncertain whether elevated androgen levels directly influence the developing embryo, given the placenta’s ability to convert androgens into estrogens. The activity of fetal adrenal glands or ovaries, which are capable of synthesizing androgens, might also play a potentially significant role [24]. In a mouse model, elevated AMH levels during pregnancy were found to reduce placental aromatase activity, leading to the masculinization of the exposed female fetus and the development of a PCOS-like reproductive and neuroendocrine phenotype in adulthood [22]. This risk of PCOS development is likely increased by impaired fetal nutrition during pregnancy, although the results of studies in this area remain inconclusive [25,26].

#### 2.1.3. Postnatal and Environmental Contributors

Various postnatal factors contribute to the development of PCOS, particularly in predisposed individuals. The most commonly observed are insulin resistance and obesity, strongly affected by lifestyle choices. Hyperinsulinemia increases serum free testosterone levels by reducing the production of hepatic sex hormone-binding globulin (SHBG), stimulates androgen production triggered by LH and insulin-like growth factor 1 (IGF-1), and enhances IGF-1 bioactivity by suppressing IGF-binding protein production [27]. Weight gain exacerbates the symptoms of PCOS, as women with a more severe PCOS phenotype are more frequently affected by obesity [3,28]. Obesity is a common feature of PCOS; however, a significant proportion of affected women are not obese [29]. Insulin resistance is common in lean women with PCOS [30]. A high carbohydrate intake is frequently recognized as a factor that can worsen PCOS presentation [31], although diet alone is unlikely to be the cause of the condition.

Other modifiable factors associated with PCOS include smoking, blood lipid levels, and alterations in the microbiota [15]. Smoking is associated with increased levels of free testosterone and fasting insulin, thereby driving the progression of the disease [32]. An increasing body of evidence suggests that environmental toxins, particularly specific endocrine-disrupting chemicals (EDCs), are associated with PCOS. Women with PCOS were found to have higher serum levels of perfluorinated compounds, polychlorinated biphenyls, pesticides, polycyclic aromatic hydrocarbons, and bisphenol A compared to healthy controls [33,34,35]. Low socioeconomic status is linked to the development of PCOS, as it is a risk factor for other contributors to the condition, such as poor diet, exposure to environmental pollutants, and obesity [36].

### 2.2. Hormonal Imbalance in PCOS

These diverse causes ultimately lead to the development of hormonal imbalances characteristic of PCOS, which are referred to as a vicious cycle due to their self-perpetuating mechanism [37]. The key hormones implicated in the pathophysiology of PCOS include androgens, hormones of the hypothalamic–pituitary–ovarian axis, and insulin. It is not easy to determine which hormone dominates in PCOS dynamics; however, ovarian hyperandrogenism is often highlighted as playing a key role. This condition arises due to primary steroidogenic hyperactivity, which disrupts the normal functioning of the ovaries [38]. One of the possible mechanisms may be the augmented transcription of the CYP17A1 and CYP11A1 genes, which are involved in androgen biosynthesis [12].

The source of androgens in PCOS, however, is more complex and diverse. Ovarian hyperandrogenism in PCOS can be directly demonstrated using the GnRH agonist test with leuprolide as a GnRH analog or the human chorionic gonadotropin (hCG) test with hCG as an LH analog [39]. 17-Hydroxyprogesterone (17-OHP) hyperresponsiveness is observed in most women with PCOS (67%), indicating a typical functional ovarian hyperandrogenism (FOH) [38]. In other cases, hyperandrogenism can be indirectly demonstrated using the dexamethasone androgen-suppression test (DAST), which reveals elevated testosterone levels [40]. This group includes cases of atypical FOH (20%) [38]. Additionally, there is a group of cases of isolated primary functional adrenal hyperandrogenism (FAH) (5%) [38], which can be identified through the adrenocorticotropic hormone (ACTH) test by measuring dehydroepiandrosterone (DHEA) levels [40]. In the remaining cases (8%), where all test results are negative, hyperandrogenism may result from obesity or have an idiopathic origin [38,40].

Another typical hormonal disturbance in PCOS is an increased release of LH relative to FSH [12]. The increased frequency and amplitude of pulsatile GnRH secretion selectively elevate LH while suppressing FSH release [41]. Additionally, high testosterone blunts the GnRH pulse generator’s sensitivity to estradiol and progesterone feedback, while tonic estrogen feedback further contributes to the increase in LH pulses [42]. LH stimulates ovarian theca cells to produce testosterone, which is only partially converted to estradiol in granulosa cells due to low FSH levels. Pituitary LH secretion is necessary to sustain the ovarian androgen excess, but it is probably not sufficient on its own to cause it [38].

In the metabolic phenotype of PCOS with high insulin resistance, all these disturbances are even more pronounced, as insulin stimulates androgen synthesis and increases its free fraction by reducing SHBG production. Despite the commonly observed high tissue insulin resistance in PCOS, the ovary paradoxically remains sensitive to insulin. Hyperinsulinemia further amplifies the stimulatory effects of LH on the ovaries [43,44].

In PCOS, serum AMH levels are elevated, as it is produced by granulosa cells of preantral and small antral follicles in proportion to their number. AMH in serum is strongly correlated with the number of early antral follicles [45]. The correlation between PCOM and serum AMH suggests, according to the American Society for Reproductive Medicine (ASRM) [4], that AMH measurement could be utilized to define PCOM in adults. In animal studies, AMH has been shown to activate GnRH neuron firing, potentially contributing to the vicious cycle of PCOS [46].

The hormonal disturbances in PCOS typically involve estrogen and progesterone. The persistent elevation in estrogen levels is primarily driven by excess androgens, which are partially converted to estrogens in the ovary or adipose tissue, as well as by infrequent or absent ovulations [47]. The lack or scarce formation of the corpus luteum is also a major cause of progesterone deficiency in PCOS [48]. It is common to observe elevated estrogen levels unopposed by progesterone, which can be referred to as relative hyperestrogenism. This phenomenon occurs due to the presence of immature ovarian follicles that produce estrogen but fail to mature and undergo ovulation. As a result, the effect of the produced estrogen is not counterbalanced by progesterone, which is low due to the lack of ovulation. This imbalance leads to the aforementioned irregular menstrual cycles, endometrial hyperplasia, and, ultimately, may contribute to the development of endometrial cancer [49,50].

Elevated estrogen levels and low progesterone concentrations are known to modulate immune system activity, which may contribute to heightened immune reactivity [51]. This hormonal imbalance is thought to drive the excessive activation of the immune system, potentially leading to the chronic low-grade inflammation often observed in women with PCOS [52]. Such immune system activation could explain the increased prevalence of various autoantibodies in this population [51]. Autoantibodies targeting ovarian tissues or hormone receptors, such as gonadotropin-releasing hormone receptor-activating autoantibodies, could disrupt normal hypothalamic–pituitary–ovarian axis function [53,54]. However, a critical question remains unanswered: are these autoantibodies merely a consequence of the altered hormonal environment, or could they actively participate in driving the pathogenesis of PCOS? We will strive to address this question in the subsequent sections of this paper.

## 3. Autoantibodies in the Pathogenesis of PCOS—Current Evidence

**Figure 2:**
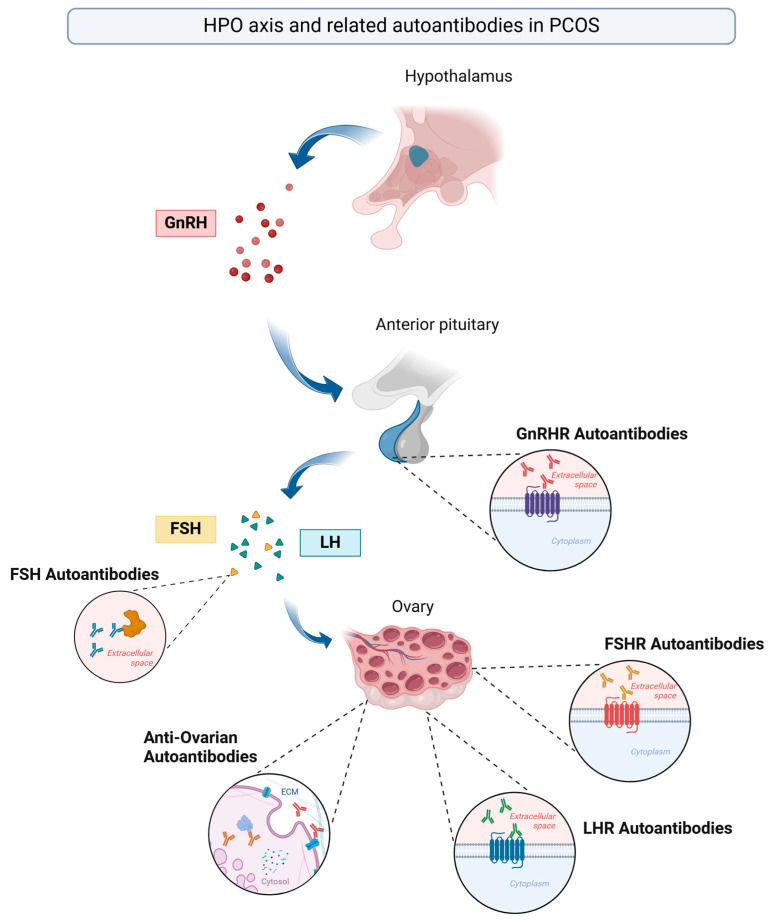
The hypothalamic–pituitary–ovarian (HPO) axis, which plays a central role in regulating ovarian function, is dysfunctional in women with polycystic ovary syndrome (PCOS), contributing to the hormonal imbalances seen in the condition. Recent studies have begun to investigate the role of autoantibodies targeting hormones, hormone receptors of the HPO axis, and ovarian tissue in women with PCOS. ECM—extracellular matrix; FSH—follicle-stimulating hormone; FSHR—follicle-stimulating hormone receptor; GnRH—gonadotropin-releasing hormone; GnRHR—gonadotropin-releasing hormone receptor; LH—luteinizing hormone; LHR—luteinizing hormone receptor. Created with Biorender.com.

Autoantibodies have been increasingly recognized as potential contributors to the pathogenesis of PCOS, particularly through their effects on key hormonal pathways. Emerging evidence suggests that immune-mediated mechanisms targeting FSH, its receptor, gonadotropin-releasing hormone receptor (GnRHR), and ovarian antigens may play a role in ovarian dysfunction, hyperandrogenism, and infertility associated with PCOS [14,53,54,55] (Figure 2).

### 3.1. Anti-FSH Autoantibodies

The dysregulation of the HPO axis in PCOS may be influenced by FSH and its receptor [56]. FSH and its signaling system play a pivotal role in normal reproductive function, regulating processes such as follicular maturation and ovulation [57]. Mutations in human FSH and its receptor have been linked to altered ovarian responses to the hormone, which can lead to varying degrees of impaired reproductive function [58]. It was suggested that FSH antibodies could be associated with a diminished ovarian response to gonadotropins [59].

In a study investigating different isotypes of anti-FSH antibodies in women with PCOS, a logistic regression analysis, adjusted for age, revealed that anti-FSH immunoglobulin A (IgA) antibodies were identified as significant risk factors for PCOS (adjusted odds ratio [OR] 2.15; *p* = 0.015) and endometriosis (adjusted OR 2.00; *p* = 0.002). Notably, neither anti-FSH immunoglobulin G (IgG) nor immunoglobulin M (IgM) levels were found to be correlated with the development of these diseases. The high levels of IgA antibodies observed in these patients may reflect an upregulation of the immune response to FSH in seminal fluid, potentially related to a mucosal immune response [55].

Further investigations explored whether serum anti-FSH antibodies targeted the immunodominant epitope of the beta-chain of FSH, known as V14D, which contains a “cysteine noose” crucial for FSH receptor binding. The study found a strong correlation between antibodies against whole FSH and V14D, suggesting that anti-FSH antibodies may target a key functional region of the hormone, potentially interfering with receptor binding in a neutralizing manner [55]. Clinically, anti-V14D IgA was identified as a risk factor for both PCOS (adjusted OR 24.68; *p* = 0.015) and endometriosis (adjusted OR 14.91; *p* = 0.004). Anti-V14D IgG was also linked to an increased risk for endometriosis (adjusted OR 6.03; *p* = 0.028) and a potential risk for PCOS (adjusted OR 6.13; *p* = 0.116), while IgM did not show a significant association [55]. These findings suggest that neutralizing anti-V14D antibodies could modulate FSH receptor binding, contributing to ovarian pathology and infertility in PCOS and endometriosis. However, it remains unclear whether these antibodies are a consequence of ovarian damage or play a primary role in infertility development [55].

### 3.2. Anti-FSHR and Anti-LHR Autoantibodies

The hypothesis that autoantibodies to the follicle-stimulating hormone receptor (FSHR) or luteinizing hormone receptor (LHR) are prevalent in PCOS was tested in only one study. Despite analyzing a relatively large sample (550 women with PCOS and 430 controls), the study found a low prevalence of these autoantibodies in both controls and women with PCOS. The authors concluded that FSHR and LHR could be potential autoantigens in humans. However, based on these findings, it is unlikely that autoimmunity to FSHR or LHR is a common cause of hyperandrogenism or ovulatory dysfunction in PCOS [60].

### 3.3. Anti-Ovarian Autoantibodies (AOAs)

Investigations into the presence of anti-ovarian autoantibodies (AOAs) in women with PCOS have also been undertaken [14]. Premature ovarian failure (POF), a condition with a complex etiology, is partially attributed to autoimmune mechanisms, as evidenced by the presence of autoantibodies against ovarian antigens [61,62]. A significant correlation has been reported between elevated anti-ovarian antibody levels and the number of in vitro fertilization (IVF) cycles, possibly due to antigen exposure from repeated ovarian punctures and microtrauma [63]. However, studies assessing AOAs in women with PCOS have yielded conflicting results. According to Fénichel et al., mean levels of AOAs were significantly higher in women with PCOS than in the control group across all studied isotypes (IgG, IgA, IgM). Positive AOAs in at least one isotype were detected in 15 (44%) of 34 women with PCOS [64]. Similarly, another study confirmed these results and found that serum AOA levels were significantly higher in cases than in controls, with a significant negative correlation between AOA and serum testosterone levels in the PCOS group [65]. In addition, higher levels of AOAs have been noted in women with PCOS with a diminished ovarian reserve compared to women with PCOS with a normal ovarian reserve [66]. Several smaller studies have also detected these autoantibodies in a limited number of women with PCOS [67,68]. However, the functional role of these autoantibodies remains unclear; it is unknown whether they act as neutralizing antibodies or reflect a secondary immune response to ovarian damage. On the other hand, according to a different study, ovarian autoimmunity was not associated with PCOS, as the frequency of ovarian antibodies was similar (25%) in both the control and PCOS groups [69]. By comparison, AOAs were detected in only 1 out of 31 women with PCOS in another study [70]. The discrepancy in the results of various studies assessing the presence of AOAs in patients with PCOS may be attributed to several factors, with the use of different antigenic substrates being one of the most important contributors. Studies that evaluate only one target antigen may fail to identify women with ovarian autoimmunity [65].

### 3.4. GnRHR Autoantibodies

In recent years, growing evidence has suggested the involvement of GnRH receptor-activating autoantibodies in the pathogenesis of PCOS, similar to what is observed in functional autoantibody diseases [71]. This finding is especially relevant given the predominant hypothesis linking GnRH dysregulation to the development of PCOS [72]. In PCOS, the pulsatile secretion of GnRH is disrupted, leading to altered LH and FSH secretion, and as a result, one of the key consequences is elevated testosterone levels, a common characteristic of PCOS [73]. Consequently, GnRH acts as the central ovarian function regulator, controlling steroidogenesis, folliculogenesis, and ovulation [74]. Any dysfunction in the GnRH hypothalamic ligand or the GnRHR pathway is therefore closely associated with fertility disturbances [75]. The following question remains: what causes the disrupted GnRH secretion? The GnRH receptor belongs to the seven-transmembrane G protein-coupled receptor (GPCR) family [76]. It is well established that autoantibodies targeting GPCRs (preferentially, the second extracellular loop (ECL2)) can induce functional alterations in cellular signaling pathways, as in Graves’ disease [77] or Chagas disease, where autoantibodies produced in response to *Trypanosoma cruzi* infection cross-react with GPCRs such as β1-adrenergic and muscarinic receptors, leading to the chronic activation of these receptors, cardiac dysfunction, and the development of Chagas cardiomyopathy [78]. Unlike natural physiological ligands, these autoantibodies do not induce receptor desensitization, leaving cells vulnerable to persistent GPCR overstimulation and its pathological consequences [13,71]. Several researchers have investigated whether activating autoantibodies against GnRHR could underlie the development of PCOS. This theory is particularly intriguing as it is supported by both clinical and preclinical studies.

A 2020 study was the first to investigate and show significantly higher levels of activating autoantibodies (AAbs) to the second extracellular loop (ECL2) of gonadotropin-releasing hormone receptor (GnRHR-ECL2-AAbs) in individuals with PCOS compared to both controls with ovulatory infertility and a group of healthy controls [13]. The potential of circulating GnRHR-AAbs to activate the receptor was tested by researchers using purified IgG from a subgroup of patients with PCOS in a cell-based GnRHR assay. A clear dose-dependent activation was observed with both natural GnRH and the synthetic analog leuprolide. Similar results were found when using IgG from patients with PCOS. The activation induced by this IgG was blocked by the GnRHR antagonist cetrorelix, allowing an estimation of the receptor-specific activity of the autoantibodies. In contrast, cetrorelix did not reduce the GnRHR-AAb activity in IgG from controls with ovulatory infertility, suggesting a lack of significant autoimmune activation of the receptor in this group. These results show that these autoantibodies do not inhibit, but rather stimulate, GnRHR activity, which may lead to a possible increase in the pulsatile secretion of GnRH and disturbances in hormonal regulation characteristic of PCOS [13]. These promising findings served as the basis for further research into understanding the role of GnRH autoantibodies in the pathogenesis of PCOS.

A major limitation of the previously described study was its small sample size; the hypothesis was subsequently tested on a larger cohort of patients with PCOS (200) and control subjects with unexplained ovulatory infertility (200) [54]. The baseline GnRHR-AAb activity was significantly higher in the PCOS group compared to the controls (3.66-fold vs. 3.45-fold increase over buffer baseline; *p* = 0.0017). After the addition of cetrorelix, GnRHR-AAb activity significantly decreased in the PCOS group from a 3.66-fold to 3.17-fold increase over buffer baseline (*p* < 0.0001), while in the control group, it remained largely unchanged. The reduction in GnRHR-AAb activity occurred in the majority of patients, with 72% of those with PCOS showing a decrease. Finally, when comparing the GnRHR-AAb activity after the addition of cetrorelix, statistically significant differences were observed between the groups [54]. The results of the study strongly support the role of autoantibodies in the development of PCOS.

Another interesting perspective on GnRHR autoantibodies is based on their relationship with LH, testosterone, and the proinflammatory cytokines interleukin-2 (IL-2), interleukin-6 (IL-6), interferon gamma (IFN-γ), and tumor necrosis factor alpha (TNF-α) [71]. Not only was increased GnRHR-AAb activity observed, along with the expected inhibitory effect of cetrorelix, in the PCOS group, but a statistically significant correlation was also found between GnRHR-AAb activity and both testosterone and proinflammatory cytokines. Interestingly, in the case of the correlation between GnRHR-AAb activity and proinflammatory cytokines, a similar linear relationship was observed for all studied molecules, suggesting that inflammation may be linked to GnRHR-AAbs. The original study’s authors propose two explanations for this phenomenon. Firstly, GnRH and its agonists have been shown to induce a proinflammatory Th1 shift, stimulating IFN-γ production, likely via GnRHR activation in immune cells. Human peripheral blood mononuclear cells (PBMCs) have been shown to express both GnRH and GnRHR mRNAs, identical to their hypothalamic and pituitary counterparts. In vitro PBMC stimulation with either GnRH or its synthetic analogs increased interleukin-2 receptor gamma-chain (IL-2Rγ) mRNA expression in these cells, suggesting a direct role for GnRH in immune cell activation and modulation [79]. This supports the hypothesis that GnRHR signaling outside the classical reproductive axis may contribute to systemic immune responses, and a similar effect could be expected for GnRHR autoantibodies. Secondly, given the interplay between androgens, adipose tissue, and inflammation in PCOS, GnRHR autoantibodies may exacerbate inflammation by disrupting adipokine balance through hyperandrogenism [71].

Establishing an autoimmune etiology for a disease requires not only identifying a specific autoantigen and immune response but also reproducing the disease in an experimental model [80]. An autoimmune PCOS model was successfully developed by immunizing rats with a synthetic peptide derived from the GnRHR-ECL2 sequence—identical to the epitope targeted by GnRHR autoantibodies in humans with PCOS [53].

The induction of GnRHR-AAbs led to changes in the animal model mirroring PCOS-related alterations in humans, including increased LH pulsatility, elevated testosterone levels, disrupted estrous cycles, increased atretic follicles, and the activation of insulin signaling in the pituitary and ovary. Insulin signaling-related genes—IRS2, PI3K, and GLUT1—were upregulated in the pituitary, while IRS1, PI3K, and GLUT1 showed increased expression in the ovary. Histological analysis revealed a thickened theca cell layer and a thinner granulosa cell layer in ovarian follicles, potentially enhancing androgen production while impairing its conversion to estrogen, ultimately contributing to ovarian dysfunction. The presence of functional GnRHR autoantibodies was also linked to elevated insulin levels, suggesting their role in disease pathogenesis [53].

Based on these findings, targeting GnRHR autoantibodies has been proposed as a potential therapeutic approach for PCOS. The same research team proposed the use of retro-inverso peptides, in which L-amino acids are replaced by D-amino acids in a reversed sequence, preserving the structure and antigenicity of the parent L-peptide while reducing susceptibility to proteolytic degradation [81]. These peptides could serve as proteolytically stable decoy molecules for GnRHR-AAbs. The researchers identified a dominant functional epitope for GnRHR autoantibodies in PCOS and demonstrated the effective inhibition of GnRHR-AAb activity in a cell-based bioassay using the epitope-mimicking retro-inverso peptide inhibitor d-CHTVCQSF [82]. Research in this area is ongoing, aiming to further explore the therapeutic potential of targeting GnRHR autoantibodies in PCOS.

Although several studies support the role of autoantibodies in the development of PCOS in both human and animal models, some conflicting reports have also emerged. The results of a study conducted on a large cohort of patients (n = 1051) were, unfortunately, not as promising. GnRHR-AAbs were detected in one patient from the control group (0.25%) and two patients from the PCOS group (0.31%), with only 12 results slightly exceeding the cutoff for a positive outcome [78]. This casts doubt not only on the hypothesis regarding the role of these autoantibodies in the pathogenesis of PCOS but, more importantly, on whether GnRHR-AAbs are in any way relevant in the context of PCOS.

Table 1 and Table 2 summarize the discussed studies regarding autoantibodies targeting components of the HPO axis in PCOS.

### 3.5. Beyond Autoantibodies: Role of T-Cell-Mediated Immunity

Despite ongoing efforts to characterize the role of autoantibodies in PCOS, other branches of the immune system may also be involved. T-cell-mediated mechanisms, particularly those involving regulatory T cells (Tregs), have been implicated in experimental models. In one study, ovarian cyst formation was observed in mice following estrogen-induced thymic disruption. Notably, mice that underwent thymectomy prior to estrogen exposure did not develop cysts, suggesting that the absence of functional Tregs is a prerequisite for cystogenesis. The authors proposed that this effect is likely due to an estrogen-induced increase in thymic vascular permeability, which interferes with the final stages of Treg development [83]. Moreover, prenatal exposure to excessive steroid levels—such as estrogen or dietary phytoestrogens—may have lasting effects on thymic function, leading to impaired immune regulation and contributing to the pathogenesis of PCOS [84]. These findings raise the possibility that T-cell-driven immune dysregulation may contribute to the development of PCOS, potentially acting independently of, or in synergy with, autoantibody-mediated mechanisms.

## 4. Is There a Place for an Autoimmune Cause of PCOS?

Recent discoveries regarding the involvement of the immune system, including autoantibodies, in the development of PCOS further complicate the heterogeneous picture of this syndrome; its full pathogenesis model remains unknown. It is essential to constantly improve our understanding of the biological causes of PCOS in terms of genetics, epigenetics, and immunology, allowing the development of targeted therapies. There is increasing evidence for different etiologies of PCOS with a distinct metabolic and reproductive phenotype [12]. We may suspect that a portion of PCOS cases may have an autoimmune cause, as indicated by the presence of autoantibodies directed against components of the HPO axis and interfering with their functional domains, such as GnRHR-ECL2-activating autoantibodies [13,54] or anti-FSH autoantibodies targeting the V14D epitope [55]. According to these findings, these patients may need to be treated differently. Most evidence supports the significance of GnRHR autoantibodies in both preclinical and clinical studies, as well as the efficacy of retro-inverso peptides in inhibiting GnRHR activity [82]. Nevertheless, we still do not know whether all these discoveries will be able to contribute to the targeted treatment of some “autoimmune” PCOS cases. A portion of available studies do not support the hypothesis of a higher prevalence of antibodies to the HPO axis in PCOS [69,78] and indicate an overall higher prevalence of autoantibodies in PCOS [51,85,86,87], which may be related to other autoimmune diseases or chronic low-grade inflammation. Paradoxically, these two observations may not be mutually exclusive. PCOS may have an autoimmune component in its pathogenesis, but it may also aggravate the production of various autoantibodies.

## Figures and Tables

**Table 1:** Summary of clinical studies on autoantibodies related to the hypothalamic–pituitary–ovarian (HPO) axis in women with polycystic ovary syndrome (PCOS).

Autoantibody	Study	Year	Results	Conclusions
GnRHR-AAbs	Kem et al. [13]	2020	OD (405 nm) value (exact values not provided) *p* = 0.000036	GnRHR-ECL2-AAbs are significantly elevated in patients with PCOS compared with healthy controls and controls with ovulatory infertility. The GnRHR antagonist cetrorelix significantly suppressed the elevated GnRHR activity induced by IgG from patients with PCOS.
	Weedin et al. [54]	2020	PCOS 3.66 ± 0.84 vs. controls 3.45 ± 0.99 GnRHR activity (fold increase over buffer baseline) adjusted ^1^ *p* = 0.0029	The baseline GnRHR-AAb activity level was significantly higher in patients with PCOS than in control subjects. The addition of cetrorelix resulted in the significant suppression of AAb activity levels in patients with PCOS as a group, whereas control subjects were unaffected.
	Li et al. [71]	2021	PCOS 39% vs. controls 0% *p* < 0.01 GnRHR activation (fold increase over buffer baseline; exact values not provided) *p* < 0.01	Serum GnRHR-AAb activity and positivity were significantly higher in patients with PCOS than in normal controls. Elevated GnRHR-AAb activity was associated with increased testosterone and proinflammatory cytokines (IL-2, IL-6, IFN-γ, TNF-α) in PCOS.
	Sattler et al. [78]	2021	PCOS 2.0% vs. controls 0.5% *p* > 0.05	Natural GnRHR-AAbs are present in a very small fraction of adult control subjects and subjects with PCOS of European decent. The results do not support the hypothesis that GnRHR constitutes a relevant autoantigen in PCOS.
anti-FSH	Haller et al. [55]	2005	anti-FSH-IgA level adjusted ^2^ OR 2.15 *p* = 0.015 anti-V14D-IgA level adjusted ^2^ OR 24.68 *p* = 0.015	The model showed anti-FSH IgA and anti-V14D IgA as significant risk factors for PCOS and endometriosis. Naturally occurring anti-FSH IgA could be a marker for infertility in ovarian disorders.
anti-FSHR	Schniewind et al. [60]	2018	PCOS 2.0% vs. controls 0.9% *p* > 0.05	The prevalence of anti-FSHR is low, both in control subjects and in women with PCOS. It is therefore unlikely that autoimmunity to FSHR constitutes a frequent cause of hyperandrogenemia or ovulatory dysfunction in PCOS.
anti-LHR	Schniewind et al. [60]	2010	PCOS 0.4% vs. controls 1.2% *p* > 0.05	The prevalence of anti-LHR is low, both in control subjects and in women with PCOS. It is therefore unlikely that autoimmunity to LHR constitutes a frequent cause of hyperandrogenemia or ovulatory dysfunction in PCOS.
AOAs	Shoukry et al. [65]	2020	PCOS 7.4 (6.7–8) ^3^ vs. controls 6.6 (6.5–7.3) ^3^ IU/mL *p* = 0.001	The presence of an autoimmune response against ovarian tissue in female patients with PCOS suggests a potential autoimmune mechanism in the pathogenesis of the condition.
	Fénichel et al. [64]	1999	OD value (exact values not provided) IgG *p* < 0.0001 IgA *p* < 0.003 IgM *p* < 0.0003	High concentrations of AOAs found in a group with PCOS suggest that an immune reaction is associated with PCOS. Positive AOAs for at least one isotype were present in 15 (44%) of 34 women with PCOS.
	Luborsky et al. [69]	1999	PCOS 25.0% vs. controls 19.0% *p* > 0.05 PCOS 0.52 (0.15–1.46) ^4^ vs. controls 0.47 (0.24–0.93) ^4^ OD (405 nm) *p* = 0.845	The frequency of AOA was similar among the controls and those with PCOS. Thus, the prevalence of ovarian antibodies in patients with PCOS is not significantly different to in controls.

Percentage values refer to antibody positivity prevalence; numerical values are given as mean ± standard deviation, unless otherwise specified; values are given in the order of PCOS vs. controls. Anti-FSH—antibodies against follicle-stimulating hormone; anti-FSHR—antibodies against follicle-stimulating hormone receptor; anti-LHR—antibodies against luteinizing hormone receptor; anti-V14D IgA—IgA antibodies against 78–93 region (V14D) of the human FSH β-chain; AOAs—anti-ovarian antibodies; GnRHR—gonadotropin-releasing hormone receptor; GnRHR-ECL2-AAbs—activating autoantibodies (AAbs) to the second extracellular loop (ECL2) of gonadotropin-releasing hormone receptor (GnRHR); IFN-γ—interferon gamma; IL-2—interleukin-2; IL-6—interleukin-6; IU/mL—international units per milliliter; OD—optical density; OR—odds ratio; PCOS—polycystic ovary syndrome; TNF-α—tumor necrosis factor alpha. ^1^ Adjusted for pair differences in age, body mass index, and anti-Müllerian hormone; ^2^ adjusted for age; ^3^ values given as median (interquartile range); ^4^ values given as mean (range of values).

**Table 2:** Short synthesis and interpretation of study results on autoantibodies related to the hypothalamic–pituitary–ovarian (HPO) axis in women with polycystic ovary syndrome (PCOS).

Autoantibody	Autoantibody Level in Women with PCOS	Summary of Study Results
GnRHR-AAbs	Probably increased	The vast majority of studies show increased levels of autoantibodies in women with PCOS, and the importance of autoantibodies has been demonstrated in preclinical studies [13,53,54,71,78].
AOAs	Possibly increased	The results are heterogeneous, but many studies show higher levels of autoantibodies in women with PCOS [64,65,69,70].
anti-FSH	Possibly increased	There is little evidence, but what exists does suggest a higher level of autoantibodies in women with PCOS [55].
anti-FSHR	Possibly not increased	There is little evidence, but what exists does not support a higher concentration of autoantibodies in women with PCOS [60].
anti-LHR	Possibly not increased	There is little evidence, but what exists does not support a higher concentration of autoantibodies in women with PCOS [60].

anti-FSH—antibodies against follicle-stimulating hormone; anti-FSHR—antibodies against follicle-stimulating hormone receptor; anti-LHR—antibodies against luteinizing hormone receptor; AOAs—anti-ovarian antibodies; GnRHR-AAbs—gonadotrophin-releasing hormone receptor-activating autoantibodies; PCOS—polycystic ovary syndrome.

## Abbreviations

The following abbreviations are used in this manuscript:

17-OHP	17-Hydroxyprogesterone
ACTH	Adrenocorticotropic hormone
AMH	Anti-Müllerian hormone
anti-FSH	Antibodies against follicle-stimulating hormone
anti-FSHR	Antibodies against follicle-stimulating hormone receptor
anti-LHR	Antibodies against luteinizing hormone receptor
anti-V14D IgA	IgA antibodies against 78–93 region (V14D) of the human FSH β-chain
AOAs	Anti-ovarian antibodies
ASRM	American Society for Reproductive Medicine
BMI	Body mass index
CVD	Cardiovascular disease
DAST	Dexamethasone androgen-suppression test
DHEA	Dehydroepiandrosterone
ECM	Extracellular matrix
EDCs	Endocrine-disrupting chemicals
FAH	Functional adrenal hyperandrogenism
FOH	Functional ovarian hyperandrogenism
FSH	Follicle-stimulating hormone
FSHB	Beta subunit of follicle-stimulating hormone
FSHR	Follicle-stimulating hormone receptor
GnRH	Gonadotropin-releasing hormone
GnRHR	Gonadotropin-releasing hormone receptor
GnRHR-ECL2-AAbs	Activating autoantibodies (AAbs) to the second extracellular loop (ECL2) of gonadotropin-releasing hormone receptor (GnRHR)
GPCR	G protein-coupled receptor
GWASs	Genome-wide association studies
hCG	Human chorionic gonadotropin
HPO	Hypothalamic–pituitary–ovarian
IFN-γ	Interferon gamma
IgA	Immunoglobulin A
IGF-1	Insulin-like growth factor 1
IgG	Immunoglobulin G
IgM	Immunoglobulin M
IL-2	Interleukin-2
IL-2Rγ	Interleukin-2 receptor gamma-chain
IL-6	Interleukin-6
INSR	Insulin receptor
IU/ml	International units per milliliter
IVF	In vitro fertilization
LH	Luteinizing hormone
LHCGR	Luteinizing hormone/choriogonadotropin receptor
LHR	Luteinizing hormone receptor
NCAH	Non-classical congenital adrenal hyperplasia
OD	Optical density
OR	Odds ratio
PBMCs	Peripheral blood mononuclear cells
PCOM	Polycystic ovarian morphology
PCOS	Polycystic ovary syndrome
POF	Premature ovarian failure
SHBG	Sex hormone-binding globulin
TNF-α	Tumor necrosis factor alpha
Tregs	Regulatory T cells
